# Pathophysiology of Primary Cilia: Signaling and Proteostasis Regulation

**DOI:** 10.3389/fcell.2022.833086

**Published:** 2022-05-11

**Authors:** Emanuela Senatore, Rosa Iannucci, Francesco Chiuso, Rossella Delle Donne, Laura Rinaldi, Antonio Feliciello

**Affiliations:** Department of Molecular Medicine and Medical Biotechnology, University Federico II, Naples, Italy

**Keywords:** ubiquitin, signaling, cAMP, PKA, autophagy, proteasome, E3 ligase

## Abstract

Primary cilia are microtubule-based, non-motile sensory organelles present in most types of growth-arrested eukaryotic cells. They are transduction hubs that receive and transmit external signals to the cells in order to control growth, differentiation and development. Mutations of genes involved in the formation, maintenance or disassembly of ciliary structures cause a wide array of developmental genetic disorders, also known as ciliopathies. The primary cilium is formed during G1 in the cell cycle and disassembles at the G2/M transition. Following the completion of the cell division, the cilium reassembles in G1. This cycle is finely regulated at multiple levels. The ubiquitin-proteasome system (UPS) and the autophagy machinery, two main protein degradative systems in cells, play a fundamental role in cilium dynamics. Evidence indicate that UPS, autophagy and signaling pathways may act in synergy to control the ciliary homeostasis. However, the mechanisms involved and the links between these regulatory systems and cilium biogenesis, dynamics and signaling are not well defined yet. Here, we discuss the reciprocal regulation of signaling pathways and proteolytic machineries in the control of the assembly and disassembly of the primary cilium, and the impact of the derangement of these regulatory networks in human ciliopathies.

## Introduction

The primary cilium is a non-motile, thin, microtubule-based organelle protruding from the apical membrane of most eukaryotic cells. Primary cilia act as antennae that receive and transmit extracellular signals into the cells, thus regulating a variety of biological functions, such as development, differentiation, growth and metabolism (Ishikawa and Marshall, 2011). The cilium consists of a basal body, a transition zone and an axoneme. The basal body derives from the differentiation of the mother centriole of the centrosome in G0 phase-arrested cells. The transition zone between basal body and axoneme acts as a gate that controls the entry and exit of cargoes within the ciliary compartment. The axoneme, also known as axial filament, is a cytoskeletal structure formed by nine doublets of microtubules surrounded by the ciliary membrane, contiguous with the cell membrane (Gerdes et al., 2009). Components of the axoneme undergo post-translational modifications that contribute to the dynamics and the stability of the cilium. The most important ciliary modification is represented by tubulin acetylation that stabilizes the axonemal structure (Wloga et al., 2017).

Primary cilia formation is strictly dependent on the cell cycle phase. Growth-arrested cells are mostly ciliated, while re-entry into the cell cycle following growth factor or hormone stimulation induces cilium resorption (Plotnikova et al., 2009). In the G0 phase of the cell cycle, vesicles generated from Golgi, named distal appendages vesicles (DAVs), are transported near the distal appendages of the mother centriole. The EH domain containing protein 1 (EHD1) promotes the fusion of DAVs in a large ciliary vesicle that incapsulates the distal appendages of the mother centriole (Lu et al., 2015). Under the ciliary vesicle, two microtubules of each centriolar triplets start to elongate, generating the ciliary axoneme. Contextually with extension of microtubules, the ciliary vesicle elongates by fusing with Rab-8 positive vesicles. The nascent cilium migrates to the plasma membrane and fuse with it, linking the two compartments (Sánchez and Dynlacht, 2016). This mechanism of cilium assembly is typical of mesenchymal cells. In epithelial cells, primary cilia formation occurs at the cells surface, in a process termed alternative route. A predominant role in this mechanism is played by the midbody, a microtubules structure surrounded by membrane, whose remnants localize at the apical surface of epithelial cells after cytokinesis. In G0 phase, when the midbody remnant (MBR) is close to the centrosome at the apical surface, patches of MBR membrane localize near the centrosome and generate the ciliary membrane (Labat-de-Hoz et al., 2021).

Since ribosomes are absent in primary cilia, ciliary proteins are synthesized into the cytoplasm and imported into the cilium mainly through the intraflagellar transport system (IFT), a multimeric complex machinery involved in anterograde/retrograde transport of cargoes along the entire length of cilium. The heterotrimeric KIF3A/KIF3B/KAP kinesin-2 is the principal motor of the IFT-B complex that regulates the movement of cargoes from the ciliary base to the tip (anterograde transport). Instead, the cytoskeletal motor protein dynein drives the IFT-A complex that controls the transport of cargoes from the tip to the ciliary base (retrograde transport) (Webb et al., 2020). Although distinct roles were initially identified, it has been recently demonstrated that IFT-A and IFT-B can participate to both anterograde and retrograde transport (Liem et al., 2012; Kobayashi et al., 2021). Mutations of genes involved in the formation, maintenance, disassembly and trafficking of primary cilium often cause developmental genetic disorders, known as ciliopathies. The principal clinical features of ciliopathies include retinal degeneration, kidney cysts formation, polydactyly, intellectual disability and skeletal defects (Badano et al., 2006; Fliegauf et al., 2007; Reiter and Leroux, 2017).

The formation and the stability of primary cilia are finely regulated by the ubiquitin-proteasome system (UPS) and the autophagy machinery, two important degradative systems operating in all eukaryotic cells. Moreover, different signaling pathways generated at- or directed to- the primary cilium operate through UPS and/or autophagy to control ciliary dynamics. Understanding how UPS and autophagy machineries influence primary ciliogenesis and identifying the mechanisms involved will lead to the identification of relevant therapeutic targets for ciliopathies and proliferative disorders, including cancer, in which ciliary pathways are often deregulated (Han et al., 2009; Moser et al., 2009; Kobayashi et al., 2017; Liu et al., 2018; Eguether and Hahne, 2018; Han et al., 2018; Peixoto et al., 2020b).

This review will focus on cilia regulation by UPS and autophagy machineries, by analyzing the dynamic interplay between these degradative systems and signaling pathways in the control of primary cilium.

## Regulation of Primary Cilia by the Ubiquitin-Proteasome System

**FIGURE 1:**
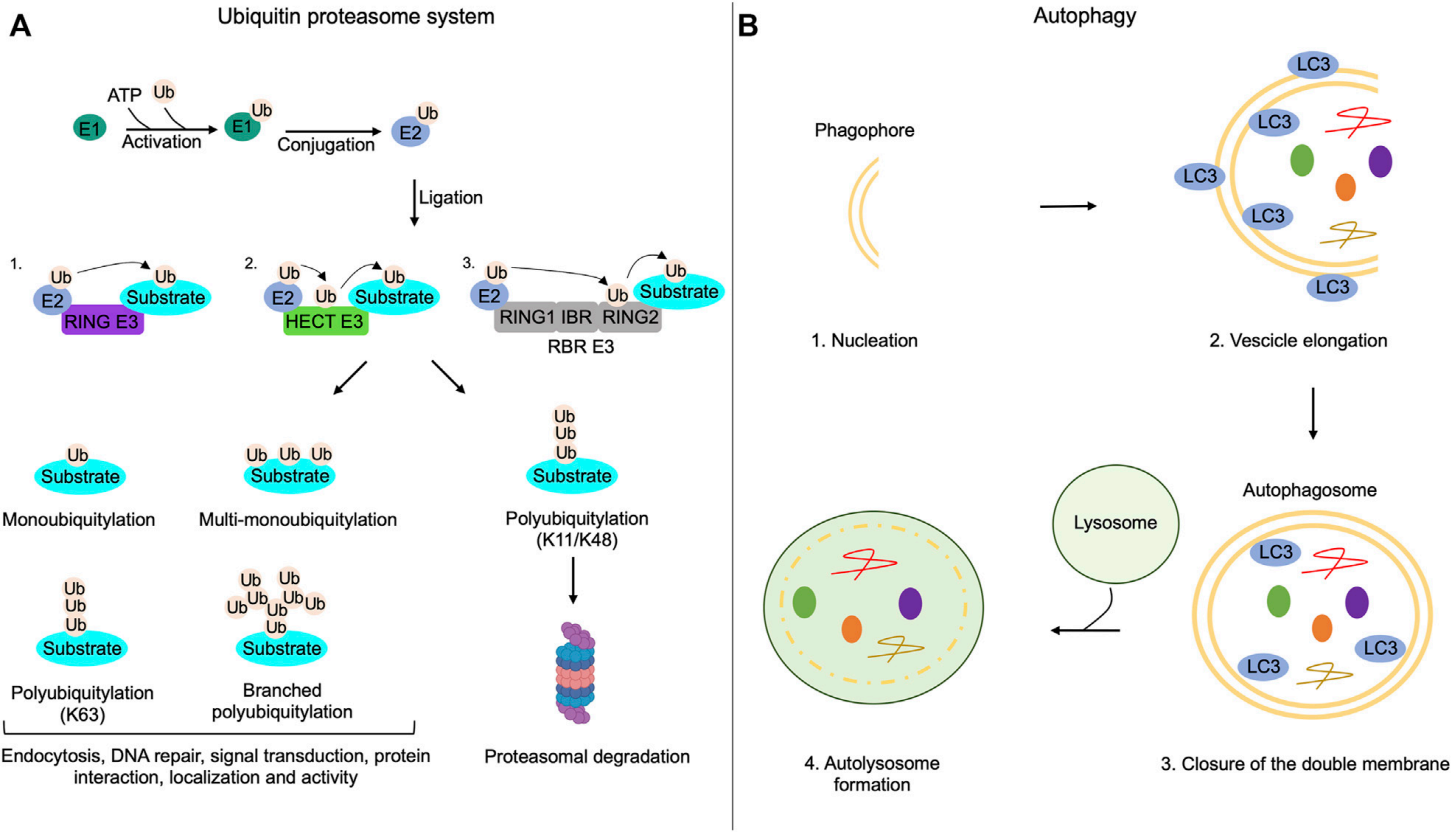
Ubiquitin-proteasome system and autophagy mechanisms. **(A)** The addition of ubiquitin moieties to substrates occurs through three sequential enzymatic reactions: binding of ubiquitin to E1 enzymes in an ATP-dependent reaction (activation); transferring of ubiquitin to E2 enzymes (conjugation); covalent attachment of the ubiquitin moiety to the target substrate by E3 enzymes (ligation). RING-E3 ligases directly transfer ubiquitin from E2 enzymes to the substrate; HECT-E3 ligases bind ubiquitin and then transfer it to the substrate; RING between RING ligases (RBR) interact with E2, bind the ubiquitin on a cysteine residue of the RING domain and then transfer it to the substrates. Substrates of the UPS can be monoubiquitylated, multi-monoubiquitylated or polyubiquitylated. Polyubiquitylations can be characterized by the addition of linear or branched ubiquitin chains. Monoubiquitylations, multi-monoubiquitylations, branched polyubiquitylations and linear polyubiquitylations that involve lysine 63 (K63) of ubiquitin regulate non-proteolytic functions of modified proteins, such as endocytosis, DNA repair, signal transduction, protein interaction, localization and activity. Polyubiquitylation that involves lysine 11 (K11) or lysine 48 (K48) on the ubiquitin is generally linked to proteolysis of the substrate through the proteasome. **(B)** Autophagy initiates with the formation of a double membrane named phagophore (nucleation); the phagophore elongates and sequestrates cargoes recognized by LC3 (vesicle elongation); the closure of the double membrane generates the autophagosome (closure of the double membrane); autophagosomes, then, fuse with lysosomes and both the inner membrane of autophagosomes and sequestered cargoes are lysed (autolysosome formation).

Proteolysis *via* the ubiquitin-proteasome system is a fundamental homeostatic mechanism that cells use to control a variety of biological functions, including differentiation, growth, development and metabolism (Dikic, 2017). The pathway works by marking with ubiquitin-like proteins (UBLs) several substrates, which will be targeted to degradation by the proteasome or to specific compartments or to relevant biological partners. In the first reaction, ubiquitin is activated through the formation of a thioester bond between its C-terminal region and the cysteine residue in the active site of E1 enzyme in an ATP-dependent reaction. Eventually, the ubiquitin moiety is transferred to the E2 conjugating enzyme. The E3 ubiquitin ligases are enzymes that mediate the transfer of ubiquitin molecules to the final target substrate. Three distinct families of E3 ubiquitin ligases have been characterized: 1) RING E3 ligases that transfer ubiquitin moieties directly from E2 to a lysine residue of the specific substrate; 2) HECT E3 ligases that bind and transfer the ubiquitin molecules from the cysteine to a lysine residue on the substrate; 3) RING-between-RING (RBR) E3 ligases with two RING domains, one interacting with the E2-ubiqutin complex and the other one binding on a cysteine residue the ubiquitin, which will be transferred to the substrate (Husnjak and Dikic, 2012; Smit and Sixma, 2014). Recently, a new class of E3 ligases, named RING-cys-relay (RCR), has been identified. RCR E3 ligases contain a RING domain that interacts with E2 enzymes and a tandem cysteine domain (TC) that transfers ubiquitin moieties to a threonine residue present on the substrate (Pao et al., 2018; Mabbitt et al., 2020). Substrates of the UPS can be mono, poly or multi-mono ubiquitylated. In the mono-ubiquitylation reaction, a single ubiquitin moiety is added to a specific lysine residue of the protein. Instead, poly-ubiquitylation requires the addition of ubiquitin chains, linear or branched, to a lysine residue. In multi-mono-ubiquitylation reactions single molecules of ubiquitin are covalently attached to different lysines in the substrate (Callis, 2014). The location of lysine residue on ubiquitin involved in poly-ubiquitylation reactions determines the fate of the target protein. Thus, ubiquitylation at lysine 11 or lysine 48 is linked to proteolysis, whereas ubiquitylation at lysine 63 mostly regulates the targeting or activity of the ubiquitylated protein. Similarly, monoubiquitylation, multi-monoubiquitylation and branched polyubiquitylation are related to non-proteolytic functions such as endocytosis, DNA repair, signal transduction, protein interaction, localization and activity (Haglund and Dikic, 2005; Sadowski et al., 2012). Proteins degradation occurs through the 26S proteasome, a multiprotein complex that catalyzes degradation of ∼80% of all cellular proteins (Bard et al., 2018). Protein ubiquitylation is a reversible process, since deubiquitylating enzymes (DUBs) can remove the ubiquitin chains from the substrates (Wilkinson, 2009) (Figure 1). A role of UPS in ciliary activities has been originally postulated and then experimentally addressed by proteomic studies and network-based approaches that identified different elements of the ubiquitin system (activating ubiquitin enzymes, E3 ligases and DUBs) as components of primary cilium (Ishikawa et al., 2012; Amato et al., 2014). In recent years, a growing list of ciliary proteins, as substrates of the ubiquitin system, supports a fundamental role of UPS in the regulation of cilium biogenesis and dynamics.

## ubiquitin-Proteasome System Promotes Cilia Assembly and Elongation

**FIGURE 2:**
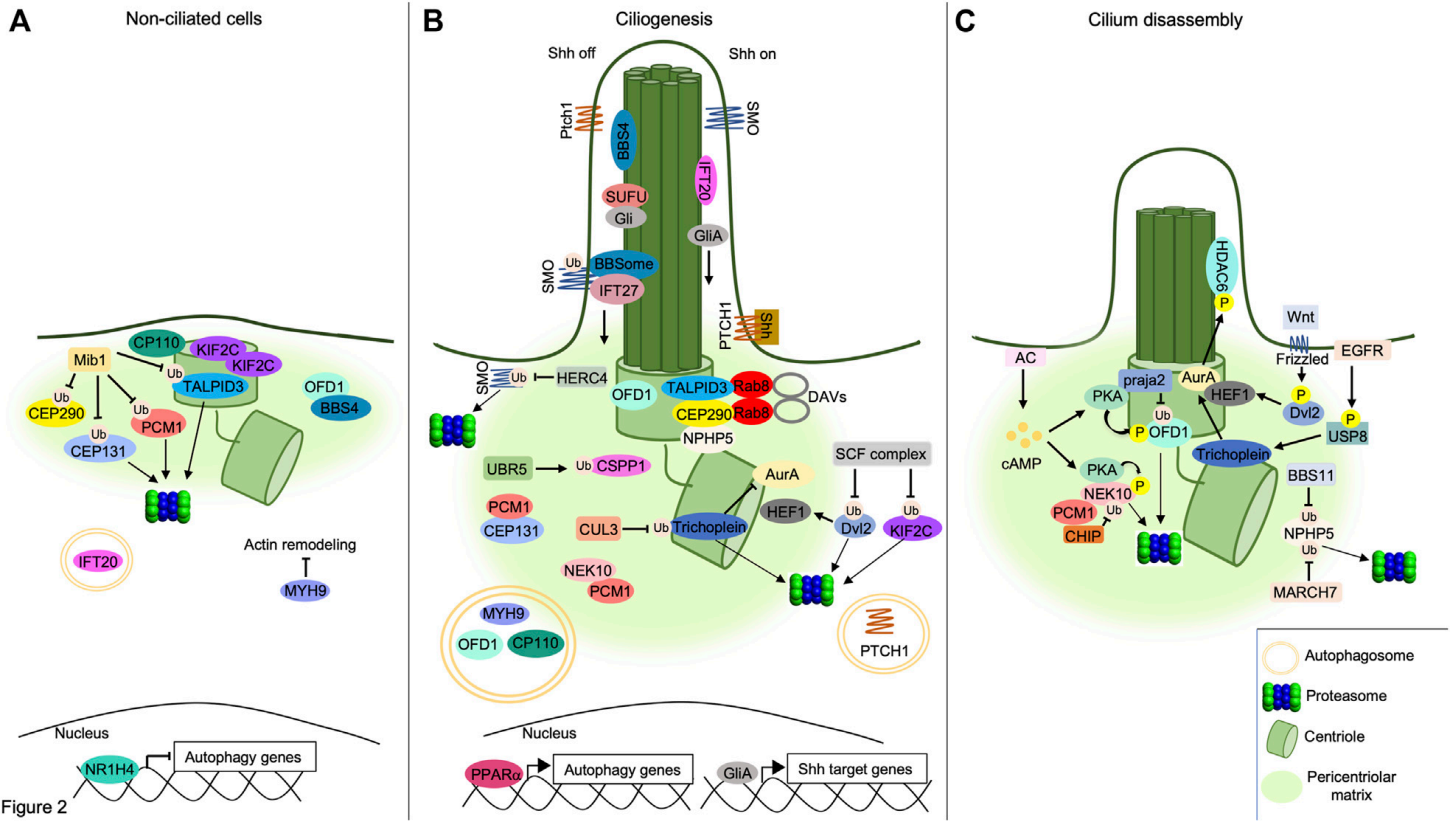
Interplay between UPS/autophagy and signaling pathways in cilia dynamics. **(A)** UPS and autophagy inhibit ciliogenesis. The E3 ligase MIB1 ubiquitylates PCM1, TALPID3 and CEP131, targeting them to the proteasome, while ubiquitylation of CEP290 by MIB1 prevents its binding to TALPID3, inhibiting the formation of DAVs. OFD1 retains BBS4 at pericentriolar satellites and prevents BBSome assembly. Accumulation of KIF2C and CP110 at basal body, inhibition of acting remodeling by MYH9 and proteolysis of IFT20 by autophagy prevent cilium elongation. The inhibition of autophagy genes by the nuclear factor NR1H4 is indicated below. **(B)** Positive role of UPS and autophagy in ciliogenesis. Centrosomal TALPID3/CEP290/Rab8 complex supports DAVs formation. UBR5 ubiquitylates and targets CSPP1 to pericentriolar satellites. Multiprotein complexes assembled by PCM1, CEP131 and NEK10 at pericentriolar matrix contribute to cilium formation. SCF E3 ligase complex ubiquitylates and degrades Dvl2, destabilizing HEF1. Similarly, CUL3 ubiquitylates and degrades Trichoplein, thus inhibiting AurA. NPHP5 accumulates at centrosome forming complexes with centrosomal CEP290 and TALPID3. Pericentriolar OFD1 is degraded through the autophagy pathway, allowing BBS4 to relocalize at ciliary compartment. Similarly, autophagic degradation of MYH9 leads to actin remodeling underlying to axoneme formation. CP110 is also degraded through autophagy leading to axoneme growth. PPARα promotes the transcription of autophagy genes. In absence of Shh ligand (Shh off), SMO is ubiquitylated by an unknown ciliary ligase and transported out of cilium by the IFT27/BBSome complex. Here, SMO is ubiquitylated and degraded by the E3 ligase HERC4. SUFU sequestrates Gli proteins within the cilium. Following binding to Shh ligand (Shh on), PTCH1 receptor exits out of cilium and is degraded through autophagy. Transport of SMO within the cilium activates GliA proteins and induces Shh-dependent nuclear gene transcription. **(C)** Cilium disassembly induced by proteolytic machineries. NPHP5 is ubiquitylated and delocalized by BBS11 or degraded by MARCH7. Phosphorylation of Dvl2 by Wnt5a stabilizes HEF1 and induces its binding to AurA. Localization of HEF1/AurA complex at basal body induces phosphorylation and activation of HDAC6. Following EGFR stimulation, USP8 deubiquitylates and activates Trichoplein which, in turn, stimulates AurA. Increase of cAMP levels by AC activates PKA. Phosphorylation by PKA primes OFD1 to ubiquitylation and proteolysis through a praja2-proteasome pathway. Similarly, PKA phosphorylates the pro-ciliogenic kinase NEK10, promoting its ubiquitylation by CHIP and consequent proteasomal degradation.

A human genome wide RNAi screening identified components of UPS that control the stability of ciliary proteins, such as IFT88 and CPAP, required for cilium assembly (Kim et al., 2016) (Figure 2). The analysis identified UBR5, an E3 ubiquitin-protein ligase and component of the N-end rule degradation pathway, as an important regulator of cilia biogenesis. Thus, UBR5 ubiquitylates the centrosome and spindle pole-associated protein 1 (CSPP1), a protein located at centrosome/centriolar satellites and ciliary axoneme, that is mutated in Joubert syndrome (Patzke et al., 2010; Tuz et al., 2014). In Human embryonic kidney cells (HEK293) and hTERT-immortalized retinal pigment epithelial cells (hTERT-RPE), the non-proteolytic ubiquitylation of CSPP1 by UBR5 regulates centrosomal localization of the protein, necessary for primary cilium assembly (Shearer et al., 2018). UPS also controls cilium assembly by promoting degradation of the inhibitors of ciliogenesis. Cullin1 (Cul1), a core component of Skip1-Cullin-F-box (SCF) E3 ubiquitin ligase complexes, ubiquitylates and degrades Dishvelled 2 (Dvl2), an inhibitor of cilium biogenesis (Cardozo and Pagano, 2004). Phosphorylation of Dvl2 by Wnt5a stabilizes the human enhancer of filamentation one protein (HEF1/Cas-L/NEDD9) and induces its binding to aurora A kinase (AurA). The HEF1/AurA complex at basal body phosphorylates and activates the Histone deacetylase 6 (HDAC6), which deacetylases tubulin favoring cilium disassembly (Pugacheva et al., 2007; Lee et al., 2012). In HEK293T and hTERT-RPE, Cul1-mediated proteolysis of Dvl2 inhibits HDAC6 activity, thus supporting ciliogenesis (Kim et al., 2021). SCF complexes also regulate cilium formation by controlling the stability of the kinesin family member 2c (KIF2C). In growing cells, KIF2C induces depolymerization of microtubules and consequent cilium disassembly (Miyamoto et al., 2015). Ubiquitin-dependent proteolysis of KIF2C by the Cullin adaptor F-Box and WD repeat domain containing 5 (FBXW5) positively contributes to the assembly of primary cilium. In human retinal pigment epithelial cells (RPE1), downregulation of FBXW5 induces accumulation of KIF2C at the basal body and impairs ciliogenesis (Schweiggert et al., 2021).

The elongation of the axonemal structure in ciliogenesis is regulated by UPS. In proliferating cells, trichoplein/mitostatin (TpMs), a keratin-binding protein often downregulated in epithelial cancers, localizes at mother and daughter centrioles and contributes to the activation of AurA (Inoko et al., 2012). Activated AurA induces deacetylation of tubulin, *via* HDAC6, and cilium disassembly (Pugacheva et al., 2007). When RPE1 cells complete the mitotic cycle, trichoplein is degraded by ubiquitin-dependent proteolysis initiated by Cullin 3 (CUL3) RING E3 ligase including also the potassium channel tetramerization domain containing 17 (KCTD17). Proteolysis of trichoplein inactivates AurA and promotes ciliary axoneme elongation (Kasahara et al., 2014). Interestingly, KCTD17-induced trichoplein degradation can be counteracted by the ubiquitin carboxyl-terminal hydrolase 8 (USP8) that deubiquitylates trichoplein. Moreover, USP8 hydrolase is stimulated and stimulates EGF receptor (EGFR) by regulating its polyubiquitylation (Berlin et al., 2010). Once activated, USP8 counteracts KCTD17-mediated ubiquitylation of trichoplein, thus suppressing primary ciliogenesis in EGF-stimulated cells (Kasahara et al., 2018).

## Dynamic Regulation of Cilium Disassembly by Ubiquitin-Proteasome System

Depending on the role of ubiquitin substrates, UPS can also inhibit the formation of primary cilia. Mindbomb E3 ubiquitin ligase protein 1 (MIB1) is an E3 ligase that inhibits initial steps of cilium formation by ubiquitylating different substrates located at centrosome/pericentriolar satellites, including pericentriolar matrix protein 1 (PCM1). This acts as a scaffold protein for components of pericentriolar satellites, including CEP131 and CEP290, thus playing a fundamental role in ciliogenesis (Woodruff et al., 2014). In human osteosarcoma cells (U2OS) and RPE1 cells, ubiquitylation of PCM1 and associated partners by MIB1 inhibits cilium formation (Villumsen et al., 2013). MIB1 also plays a role in the regulation of Rab8-containing DAVs that are essential for the transition from mother centriole to basal bodies. Thus, TALPID3 gene, mutated in a mild form of Joubert syndrome, encodes for a centrosomal protein required for ciliogenesis and Sonic Hedgehog (Shh) signaling pathway. TALPID3 localizes at distal end of centrioles where it forms a multiprotein complex including CP110, CEP290 and Rab8A. The TALPID3 complex promotes formation of ciliary vesicles at centrioles that eventually fuse with secondary vesicles to form the ciliary membrane around the assembling axoneme (Kobayashi et al., 2014). Under growing conditions in HEK293T cells, ubiquitylation of CEP290 and TALPID3 by MIB1 inhibits the DAVs formation at the centrosome. In growth-arrested RPE1 cells, PCM1 retains MIB1 at pericentriolar satellites, preventing TALPID3 ubiquitylation and leading to cilia formation (Wang et al., 2016). In HeLa cells, ubiquitylation of PCM1 by MIB1 is counteracted by the ubiquitin carboxyl-terminal hydrolase 9X (USP9X), a deubiquitylating enzyme that protects PCM1 from proteolysis, maintaining the integrity of centriolar satellites (Han et al., 2019).

UPS also controls the disassembly of primary cilia. A fundamental role in this process is played by nephrocystin 5 (NPHP5), a gene product mutated in Senior-Løken syndrome, a ciliopathy disease characterized by congenital amaurosis (Leber congenital amaurosis) and nephronophthisis (Stone et al., 2011). During interphase, NPHP5 is located at the centrosome and interacts with CEP290, positively controlling the integrity of BBSome, a conserved multiprotein complex that controls trafficking of cargoes and receptors within the primary cilium (Barbelanne et al., 2013, 2015). In G2/M phase transition in HEK293 and RPE1 cells, ubiquitylation of NPHP5 by the E3 ubiquitin ligase TRIM32/BBS11 induces delocalization of the protein from centrosome and consequent cilium disassembly. Similarly, a MARCH family member of membrane-bound E3 ubiquitin ligase (MARCH7) ubiquitylates and degrades NPHP5 promoting cilia loss. As expected, the activity of both E3 ligases, TRIM32 and MARCH7, is counteracted by the deubiquitylase USP9X. In fact, in growth-arrested conditions, USP9X is recruited at the centrosome, deubiquitylates NPHP5 and prevents its degradation and cilium disassembly (Das et al., 2017).

## Dysregulation of Ubiquitin-Proteasome System in Ciliopathy Disorders

Given the crucial role of UPS in primary ciliogenesis, it is important to consider the potential impact of UPS dysregulation in the pathogenesis of human ciliopathies. A direct link between dysfunctional UPS and ciliopathies has been established by identifying loss-of function mutations of RPGRIP1L (RPGRIP1 like) that are causally linked to Joubert syndrome and Meckel syndrome (Wiegering et al., 2018). In normal cells, RPGRIP1L protein localizes at transition zone of primary cilium and here it controls the ciliary targeting of components of the proteasome, such as PSMD2 protein (Gerhardt et al., 2015). Loss of function mutation of RPGRIP1L impairs the proteasomal activity at ciliary compartment, thus contributing to ciliopathy phenotype. Similarly, the loss of BBS4 and OFD1, two ciliopathy-related proteins, decreases localization of proteasomal subunits at the centrosome, causing the accumulation of Sonic Hedgehog and Notch signaling mediators that are normally degraded by UPS. Accordingly, the overexpression of proteasomal subunits or the chemical activation of the proteasome partially restores the signaling defects in BBS4 and OFD1 deleted Zebrafish (Liu et al., 2014). Furthermore, a genome wide RNAi screening identified the deubiquitylating enzyme USP35 as a genetic suppressor of Bardet-Biedl syndrome 4 (BBS4). Inactivation of USP35 in a zebrafish model of BBS4 ciliopathy, rescues different ciliary defects, such as impaired convergent and extension movements during gastrulation, renal tubule convolution and retinal degeneration, thus ameliorating the phenotype(s) of BBS4-depleted animals (Tsai et al., 2019).

These results suggest a primary remarkable role of UPS dysregulation in the onset of ciliopathy disorders and highlight potential therapeutic targets to restore primary cilium functions by locally modulating UPS activity.

## Autophagy Control of Ciliogenesis

Autophagy is an intracellular self-degradative process that is essential for the maintenance of the cellular energetic balance in response to nutrient stress or to eliminate dysfunctional proteins and organelles, playing a fundamental role in different physiological conditions, such as development, tissue homeostasis, metabolic adaptation and signaling, immunity, inflammation and elimination of microorganisms. Derangement of the autophagy pathway is linked to a variety of human disorders (Mizushima, 2005; Klionsky et al., 2021). Under nutrients supplementation, basal level of the autophagy machinery supports the turnover of organelles and proteins. During nutrients deprivation, autophagy is rapidly activated to maintain the appropriate energetic supply for all cellular activities (Levine and Kroemer, 2008). Autophagy is a multistep process that includes: 1) nucleation of autophagic vesicles (phagophores); 2) vesicles elongation with sequestration of cargoes; 3) closure of the double membrane; 4) fusion of formed autophagosomes to lysosomes. In the fused vesicles, both the inner membrane of autophagosomes and cargoes are enzymatically and chemically eliminated (Figure 1) (Levine et al., 2011). The autophagy machinery is driven by the sequential activation of a variety of gene products originally identified in yeast, named ATGs, that are highly conserved in mammalian genome. The initiation of autophagic process is controlled by signaling pathways involving the mechanistic target of rapamycin (mTOR kinase) and AMP-activated protein kinase (AMPK) that oppositely control autophagy through direct phosphorylation of Unc-51 Like Autophagy Activating Kinase 1 (ULK1) at distinct sites (Kim et al., 2011). For many years, autophagy has been considered as a bulk sequestration system. However, it is now well established that autophagy profoundly contributes to different aspects of cellular homeostasis by selectively removing cytoplasmic materials, such as protein aggregates, damaged organelles and invading pathogens (Zaffagnini and Martens, 2016).

Cargoes targeted to the autophagy machinery are recognized by ATG8 family members (LC3 and GABARAP proteins) through direct interaction with LC3-interacting regions (LIRs) present in a variety of target proteins or *via* receptors, such as p62 (also known as Sequestosome 1, SQSTM1), a multifunctional adapter protein that recognizes ubiquitinated cargoes and facilitates their elimination by the degradative pathway (Pankiv et al., 2007; Birgisdottir et al., 2013). Considering that both autophagy and ciliogenesis occur under conditions of nutrients deprivation, a functional link between both processes has been originally supposed and subsequently supported by several lines of evidence (Figure 2). Thus, signaling pathways activated at ciliary compartment, such as Sonic Hedgehog, stimulate the autophagy process through the regulation of autophagy-related proteins located at the base of primary cilium. Inhibiting ciliogenesis negatively impacts on the autophagy pathway. Moreover, ATG proteins have been identified as resident of the ciliary compartment and have been implicated in the regulation of ciliary signaling and activities (Pampliega et al., 2013; Yamamoto and Mizushima, 2021). In growing cells, basal activation of autophagy machinery inhibits cilia formation, at least in part, through the autophagic degradation of IFT20, a component of the IFT system involved in cilium assembly. Hence, the inhibition of the autophagy pathway induces cilia extension and signaling. In contrast, under nutrients deprivation, IFT20 protein is stabilized and accumulates in the ciliary compartment, positively contributing to ciliogenesis (Pampliega et al., 2013). This positive effect of autophagy on cilium biogenesis has been, at least in part, mechanistically linked to proteolysis of OFD1, a protein localized at the centrosome/basal body and pericentriolar satellites. In growing cells, OFD1 restraints ciliogenesis by retaining BBS4, a component of the BBSome, at pericentriolar satellites. Starvation-induced activation of autophagy machinery promotes targeted proteolysis of the pericentriolar pool of OFD1, with consequent release of BBS4 and BBSome formation, a prerequisite for the onset of ciliogenesis (Tang et al., 2013). Moreover, in serum-deprived mouse embryonic fibroblasts (MEFs), autophagy promotes primary cilia formation, at least in part, through the degradation of CP110. CP110 is a centrosomal protein that suppresses ciliogenesis by capping the distal ends of both centrioles. During starvation, the autophagic degradation of CP110 mediated by NudC-like protein 2 (NudCL2), a selective autophagy receptor at the mother centriole, induces cilium formation. This mechanism was confirmed also in Zebrafish models, where depletion of NudCL2 generates a ciliary phenotype that is rescued by CP110 depletion (Liu et al., 2021).

The differential effects of autophagy on ciliogenesis can be explained by the use of different cellular contexts and experimental models. Thus, in retinal cells, autophagy promotes ciliogenesis, whereas in renal cells it inhibits cilia elongation. In serum-deprived human kidney 2 (HK2) cells, induction of autophagy by rapamycin or Tat-Beclin1 peptide treatment significantly reduces cilia length, suggesting that autophagy inhibits cilia formation. A potential mechanism concerns VPS39, a component of the HOPS complex involved in the fusion of lysosomes with autophagosomes vacuoles. In kidney cells, VPS39 controls localization of IFT20 and OFD1 at pericentriolar satellites, negatively regulating cilia elongation. Genetic silencing of VPS39, by reducing OFD1 levels at pericentriolar satellites and promoting recruitment of IFT20 at ciliary structures, induces cilia overgrowth. This effect was reversed by concomitant activation of the autophagy machinery, supporting the notion that in renal cells autophagy prevents cilia elongation by controlling OFD1 and IFT20 localization at pericentriolar satellites (Iaconis et al., 2020). A similar role for autophagy in ciliogenesis has been described in epithelial cells of the respiratory tract. In these cells, the shortening of cilia length by cigarette smoking, a process termed “ciliophagy,” can be prevented by heterozygous knockout of the autophagic gene beclin1 (Lam et al., 2013). The autophagy-induced shortening of primary cilia is mediated by HDAC6, a deacetylating enzyme involved both, in cilia loss and maturation of autophagosome (Lee et al., 2010; Gradilone et al., 2013). Downregulation of HDAC6 restores cilia length in mice exposed to cigarette smoking (Lam et al., 2013). This mechanism has been observed also in cholangiocarcinoma cells, where the inhibition of either HDAC6 or autophagy machinery increases cilium length. Mechanistically, HDAC6-mediated deacetylation of ciliary proteins favors their ubiquitylation and consequent recognition by autophagy receptors, such as NBR1 and CALCOCO2, that target them to autophagic degradation, thus reducing cilium length (Peixoto et al., 2020a). An involvement of the autophagy machinery in the control of ciliogenesis in airway epithelial cells has been recently described. Thus, AMPK activation promotes autophagic degradation of KIF19A, a microtubule-depolymerizing kinesin located at the cilia that is required for ciliary length control, and cilia disassembly. This mechanism is counteracted by adenylate cyclase 6 (AC6) that inhibits AMPK binding to KIF19A, preventing cilia disassembly. Accordingly, AC6 knock-out airway cilia are deficient in kinesin KIF19A and show abnormal length and function (Arora et al., 2020). Altogether, these data suggest that autophagy exerts differential effects on ciliogenesis depending on cell types and players involved.

An additional layer of complexity in the interplay between autophagy and ciliogenesis is represented by myosin heavy chain 9 (MYH9)/myosinIIA, a suppressor of actin dynamics and negative regulator of primary ciliogenesis (Rao et al., 2014). During autophagy, NIMA-related kinase 9 (NEK9), by employing its LIR domain, binds MYH9 and facilitates its elimination through the autophagy machinery (Yamamoto et al., 2021). NEK9 also controls the stability of the pericentriolar pool of OFD1, most likely acting through an unidentified adaptor-independent mechanism. This hypothesis was supported by identification of LIR domains on OFD1 protein that directly interact with autophagosomal LC3/GABARAP proteins, mediating the elimination of the protein through the autophagy machinery (Morleo et al., 2021). By promoting the autophagic removal of MYH9 and OFD1 protein complexes, NEK9 restores actin remodeling and promotes cilium elongation (Yamamoto et al., 2021).

Peroxisome proliferator-activated receptors (PPARα) and nuclear receptor subfamily one group H member 4 (NR1H4) regulate ciliogenesis through autophagy. PPARα is activated under fasted conditions and positively regulates the transcription of genes involved in the autophagic pathway (Lee et al., 2014). Treatment with PPARα ligand in different mammalian cell lines induces cilia formation, both in normal and serum-deprived conditions, and these effects are abrogated in ATG7 knockout mouse embryonic fibroblasts (MEFs) (Liu et al., 2018). Conversely, NR1H4 inhibits autophagy by repressing the transcription of autophagic genes (Seok et al., 2014). As expected, treatment with NR1H4 ligand in serum-deprived cells reduces primary ciliogenesis, whereas genetic silencing of NR1H4 promotes ciliogenesis, even under normal medium conditions. These studies demonstrate that transcriptional regulation of autophagic genes by nuclear receptors PPARα and NR1H4 controls ciliary dynamics (Liu et al., 2018b).

Another important aspect of the reciprocal regulation between ciliogenesis and autophagy is represented by the primary cilia-autophagy-NRF2 (PAN) axis, originally identified in human embryonic stem cells (hESC). Thus, during neuroectodermal differentiation, the ciliogenesis leads to the activation of autophagy machinery that, in turn, inhibits the nuclear factor erythroid 2-related factor 2 (NRF2), promoting the neuroectodermal differentiation (Jang et al., 2016). The function of PAN axis has been demonstrated also in fibroblasts, in which the inhibition of ciliogenesis leads to upregulation of NRF2 activity that can be rescued by autophagy-activating mTOR inhibitors (Martin-Hurtado et al., 2019).

## Autophagy and Ciliopathies

Dysregulation of autophagy pathway can contribute to the onset of ciliopathies, thus representing a valuable therapeutic target for ciliary disorders. The pathogenic role of autophagy in human ciliopathies emerged from studies on polycystic kidney disease (PKD), the most common form of renal cystic genetic disorder associated with alterations of primary cilia (Kathem et al., 2014). In PKD, there is an impairment of autophagy machinery mostly due to altered fusion between autophagosomes and lysosomes vesicles (Belibi et al., 2011). The impaired autophagic flux has been reproduced in zebrafish mutants for PKD1, as well as in mouse and human PKD1-null kidney epithelial cells. In these models, downregulation of the core autophagy protein ATG5 increases cysts growth (Zhu et al., 2017). Conversely, activation of autophagy machinery by a specific inducer Beclin-1 peptide markedly attenuates the cystic phenotype and ameliorates the kidney function (Chang et al., 2017; Zhu et al., 2017). These data indicate that the use of chemical modulators of autophagic pathway represents a valuable strategy for the treatment of ciliopathy disorders.

Although the involvement of autophagy in the onset of autosomal dominant polycystic kindey disease (ADPKD) has been largely demonstrated, only modulators of mTOR and AMPK activity have been tested in clinical studies for the treatment of ADPKD. Rapamycin and other mTOR inhibitors are very effective in experimental studies (Tao et al., 2005; Shillingford et al., 2006, 2010; Wahl et al., 2006). However, in clinical trials they showed loss of efficacy in the progression of renal impairment (Serra et al., 2010; Walz et al., 2010; Lin et al., 2019). Preclinical studies have demonstrated also the efficiency of metformin, an activator of AMPK, in ADPKD treatment (Takiar et al., 2011; Chang et al., 2017; Pastor-Soler et al., 2022). Recently, metformin was tested also in randomized clinical trials in which it has been shown that it is safe but slightly reduces renal impairment (Seliger et al., 2018; Perrone et al., 2021).

In addition to PKD, other ciliopathies have been causally linked to altered autophagy. RPGRIP1L gene mutations are associated with different ciliopathies mostly due to impairment of protein degradation and protein processing by UPS (Gerhardt et al., 2015). However, drug-induced restoration of proteasomal activity does not completely rescue the ciliary phenotype induced by RPGRIP1L loss, suggesting that this protein works through a different mechanism in regulating ciliary activity. Evidence revealed that RPGRIP1L absence, thus, impairs autophagy at ciliary compartment by increasing the activation of MTOR complex 1 (MTORC1) pathway and the levels of OFD1 at the base of cilium. Inhibition of MTORC1 activity by rapamycin restores both the autophagic flux and the cilia length in RPGRIP1L-null MEFs, without affecting proteasomal activity. This data indicates that RPGRIP1L independently regulates both autophagic and proteasomal activities to control ciliogenesis (Struchtrup et al., 2018). A R998Q mutation of serine/threonine-protein kinase VPS15, a PI3K regulator and component of the autophagic machinery, has been identified in family members affected by shorter cilia. VPS15 forms a complex with Golgin GM130 and regulates the trafficking of intraflagellar protein IFT20 from Golgi apparatus to ciliary compartment. Mutations of VPS15 retain IFT20 at Golgi membranes, impairing IFT20-dependent transport of membrane proteins from cis-Golgi to ciliary compartment, thereby reducing cilium elongation (Stoetzel et al., 2016). The transport of IFT20 to the primary cilium is also regulated by the autophagy protein ATG16L1. Thus, following serum deprivation, ATG16L1 and IFT20 form a stable complex that is co-transported from the Golgi apparatus to the ciliary compartment. In absence of ATG16L1, IFT20 accumulates in the Golgi apparatus, causing aberrant ciliary structures (Boukhalfa et al., 2021).

A mechanistic link between human ciliopathies and deregulated autophagy machinery has been recently described. Oral-Facial-Digital type I syndrome (OFDI), a ciliopathy disorder caused by mutations of OFD1 gene, is characterized by upregulation of the autophagy pathway. Mechanistically, evidence indicates that OFD1 protein acts as an autophagy receptor that participates in an “autophagy self-regulated mechanism” that promotes autophagic elimination of ATG13, a component of the ULK1 autophagy initiation complex. By removing ATG13, OFD1 limits excessive activation of the autophagy machinery. Accordingly, inhibition of autophagy in mouse models of OFDI ameliorates polycystic kidney, a typical clinical manifestation of the disease (Morleo et al., 2021).

Autophagy alterations causing defective ciliogenesis have been correlated also to non- hereditary neurological diseases, such as focal malformations of cortical development (FMCDs). This is a pediatric intractable epileptic disorder characterized by cortical dyslamination, focal cortical dysplasia (FCD) and hemimegaloencephaly (HME). Activating somatic mutations of mTOR kinase have been identified and causally linked to FMCDs. Mechanistically, uncontrolled mTOR activation leads to defective autophagy, accumulation of OFD1 at the pericentriolar satellites and inhibition of neuronal ciliogenesis. Disrupted ciliogenesis affects Wnt pathway, essential for neuronal polarization, and leads to cortical dyslamination typical of FMCDs (Park et al., 2018). Altogether, the data indicate an important role of autophagy in ciliary dynamics and signaling, and suggest the possibility to target the autophagic machinery to treat different forms of ciliopathies.

## Crosstalk of Ubiquitin-Proteasome System, Autophagy and Ciliary Pathways

Primary cilia act as transduction hubs that transmit extracellular signals into cell body. For this function, primary cilia are considered as “the cell’s antenna” that sense, transmit and regulate signaling pathways involved in essential aspects of cellular homeostasis and organ development. Different membrane receptors, including GPCRs, and signaling molecules have been identified as resident of primary cilia (Wheway et al., 2018). Trafficking of the receptors and associated partners throughout the cilium subserves as a mechanism to finely control the rate and magnitude of cilium-based signaling pathways. Reciprocal regulation between signaling pathways, UPS and autophagy machinery in the control of ciliary dynamics has been described. Once activated, GPCRs are rapidly transported out of the cilium by the BBSome complex (Ye et al., 2018). BBSome-mediated removal of GPCRs requires K63 ubiquitylation of the receptor by a β-Arrestin-mediated mechanism (Shinde et al., 2020). The ubiquitin system, thus, acts as a general control mechanism for ciliary signaling pathways.

Sonic Hedgehog (Shh) is the most important signaling pathway operating within the primary cilium and plays a fundamental role in development, regeneration and organ homeostasis. Derangement of Shh pathway is causally associated to ciliopathy disorders and cancer (Dafinger et al., 2011; Hynes et al., 2014; Sasai et al., 2019). Shh pathway operates within intact primary cilia and most of Shh components are located within the ciliary compartment. Under resting conditions, the tumor suppressor membrane receptor Patched (PTCH1) inhibits the accumulation of smoothened (SMO), a class frizzled GPCR and component of Shh, in the ciliary compartment (Rohatgi et al., 2007). In these conditions, suppressor of fused protein (SUFU) sequestrates the Shh transcription effectors Gli proteins in the cilium. Here, at ciliary base, PKA phosphorylation primes Gli proteins processing into transcriptional repressors. The binding of Shh ligand to PTCH1 activates SMO that translocates to ciliary compartment, inducing the release of unprocessed, active Gli proteins from SUFU, allowing them to migrate into the nucleus and activate the transcription of target genes (Corbit et al., 2005; Tukachinsky et al., 2010).

UPS plays a fundamental role in the regulation of the Shh pathway at different levels. It controls PKA-dependent processing of Gli2 and Gli3 proteins in the repressive forms. Similarly, other components of Shh pathway are regulated by the ubiquitin system (Gerhardt et al., 2016). The regulation of Shh pathway by UPS also impacts on cilia dynamics. Thus, the ubiquitin carboxyl-terminal hydrolase 14 (USP14), by regulating the stability of KIF7, a member of kinesin-4 family and essential regulator of microtubule dynamics and Shh pathway, inhibits ciliogenesis and cilia elongation. Pharmacological inhibition of USP14 restores Shh activity and ciliogenesis in PKD1 mutant MEFs (Massa et al., 2019). UPS also controls the ciliary localization of SMO. In absence of ligand, ubiquitylated SMO exits from the cilium through the IFT system and BBSome complex. Inhibition of ubiquitylation allows SMO accumulation in the ciliary compartment, even in the absence of Shh ligand (Desai et al., 2020). Moreover, SMO levels are regulated by UPS. HECT and RLD domain containing E3 ligase 4 (HERC4) ubiquitylates SMO promoting its degradation through the proteasome, as well as *via* lysosomes. Shh stimulation or HERC4 inactivation inhibits HERC4/SMO complex formation and prevents SMO proteolysis, thus activating downstream pathways (Jiang et al., 2019). Deubiquitylation of SMO by the ubiquitin carboxyl-terminal hydrolase isozyme 5 (UCHL5/UCH37) also positively regulates Shh signaling (Zhou et al., 2018). All these studies suggest that targeting UPS constitutes a powerful strategy to modulate Shh pathway at the ciliary compartment, restoring deranged ciliary signaling in ciliopathy disorders.

Shh pathway is also controlled by autophagy. Following ligand stimulation, PTCH1 undergoes to polyubiquitylation and proteolysis, promoting accumulation of SMO at ciliary compartment. Blocking macroautophagy, by preventing PTCH1 ubiquitylation, reduces the transport of SMO to the cilium and impairs Shh pathway (Yang et al., 2021). Reciprocal regulation between autophagy and Shh in the control of cilium dynamics and activity has been described (Li et al., 2012; Petralia et al., 2013; Wang et al., 2013). Serum-deprived Gli2-knockout NIH3T3 cells show longer cilia and increased autophagic flux. This lengthening of primary cilia is caused by increased autophagic degradation of pericentriolar OFD1. Blocking the autophagy machinery rescues ciliary length in Gli2^-/-^ cells, indicating that Gli2 controls cilia elongation through autophagy (Hsiao et al., 2018). Furthermore, Gli2^-/-^ cells display a significant delay in cell cycle re-entry that can be rescued by concomitant downregulation of KIF3A, supporting the existence of a functional link between autophagy, cilium length and cell proliferation (Hsiao et al., 2018).

Shh can also induce ciliogenesis through a non-canonical pathway involving the SMO-mediated activation of LKB1/AMPK pathway, a positive regulator of autophagy. Thus, in LKB1 knockdown cells, stimulation with Shh fails to induce ciliogenesis and this phenotype can be rescued by treatment with autophagy activators (Akhshi and Trimble, 2021). Evidence indicates that Shh regulates early steps of autophagic process. Activation of autophagy by nutrients deprivation requires the synthesis and accumulation of components of the autophagic machinery at the base of primary cilium through a mechanism involving the IFT system and Hh signaling. Interfering with the IFT compromises autophagy and this can be rescued by Gli2 overexpression (Pampliega et al., 2013). These studies indicate that Shh pathway controls ciliogenesis and cilia dynamics by positively regulating autophagy, which in a feed-back loop controls ciliary Shh signaling. Considering that dysregulation of Shh signaling constitutes a common feature in several ciliopathies, the data strongly support the use of modulators of the autophagy pathway as therapeutic strategy for the treatment of these disorders.

UPS can also regulate ciliary pathways by modulating the stability of tyrosine kinase receptors located within the cilium, such as platelet-derived growth factor receptor *α* (PDGFRα). During cilium elongation, localization of PDGFRα at ciliary compartment is required for local activation of the receptor by PDGF-AA ligand. Once activated, ciliary PDGFRα undergoes to ubiquitylation and internalization through a mechanism controlled by ciliary IFT20/E3 ligase Cbl complexes. The receptor internalization limits the downstream activation of the cascade that might otherwise lead to overgrowth of primary cilia (Schmid et al., 2018).

## The Impact of cAMP-Regulated Proteolytic Systems on Primary Cilia

cAMP is an ancient second messenger that mediates the biological responses to a variety of hormones and neurotransmitters, controlling metabolism, differentiation, cell growth, development, and synaptic activities. The main effector of cAMP is protein kinase A (PKA). PKA is a tetrameric holoenzyme composed of two regulatory (R) and two catalytic (PKAc, C) subunits. Ligand-induced activation of dedicated GPCRs at plasma membrane activates the adenylate cyclase which in turn synthesizes cAMP. The binding of cAMP to R subunits dissociates PKA holoenzyme and releases free active C subunits. Phosphorylation of cellular substrates by C subunit controls most of the cAMP functions inside cells (Taylor et al., 2013; Newton et al., 2016; Rinaldi et al., 2019). PKA holoenzyme is compartmentalized at discrete intracellular sites by direct binding to A-Kinase Anchor Proteins (AKAPs). AKAPs operate as transduceosomes that assemble components of cAMP generating systems (receptors and adenylate cyclase), effector enzymes (PKA and Epac) and attenuating enzymes (cAMP-phosphodiesterases and protein phosphatases), creating intracellular sites where distinct signaling pathways converge and focus, optimizing the biological response to a given stimulus (Carlucci et al., 2008; Lignitto et al., 2011; Yang and McKnight, 2015; Jones et al., 2016; Rinaldi et al., 2017, 2018; Bucko et al., 2019).

Primary cilia, functioning as transduction hubs, are enriched of receptors, including GPCRs, that possess a ciliary localization sequence that allow their transport from Golgi to ciliary membrane (Berbari et al., 2008). Different components of the cAMP pathway have been identified within the primary cilium, including AKAP/PKA complexes, adenylates cyclases, phosphodiesterares (PDEs) and phosphatases (Barzi et al., 2010; Choi et al., 2011; Mick et al., 2015). Ciliary cAMP-PKA axis plays an inhibitory role in the Shh pathway finely controlling the proteasomal processing of the transcription activators Gli2 and Gli3 to transcriptional repressors (Pan et al., 2009; Chen et al., 2011; Niewiadomski et al., 2014). PKA-regulated UPS system is involved in the regulation of cilium biogenesis and dynamics (Pal and Mukhopadhyay, 2015; Bachmann et al., 2016; Tschaikner et al., 2020). Never in mitosis A (NIMA)-related kinase 10 (NEK10) is a member of mitotic kinases that regulates mitogenesis, cilium biogenesis and airway mucociliary clearance (Fry et al., 2012; Porpora et al., 2018; Chivukula et al., 2020). Genetic inactivation of NEK10 affects primary ciliogenesis in mammalian cells and medaka fish development. Moreover, germline inactivating mutations of NEK10 have been causally linked to a human ciliopathy syndrome characterized by pathological airway dilation, impaired mucociliary clearance and bronchiectasis (Chivukula et al., 2020).

An interplay between PKA, NEK10 and UPS pathway has been established. Thus, in course of cAMP stimulation, PKA phosphorylates NEK10 and induces its ubiquitylation and degradation mediated by the E3 ligase chaperone-assisted C-terminus of Hsc70-interacting protein (CHIP/Stub1). By reducing NEK10 levels, PKA-CHIP complex leads to cilia resorption (Porpora et al., 2018). This regulatory system is lost in autosomal recessive spinocerebellar ataxia-16 (SCAR16) patients fibroblasts carrying germline inactivating mutations of CHIP (Porpora et al., 2018). cAMP, acting through the UPS, also controls the stability of OFD1. In serum deprived condition, membrane stimulation of cAMP synthesis induces PKA phosphorylation of OFD1. Phosphorylated OFD1 is rapidly ubiquitylated by E3 ligase praja2 and degraded by the proteasome. Proteolysis of OFD1 markedly reduces bulk levels of the protein and impairs primary cilium elongation and medaka fish development. This mechanism is defective in an OFD1 variant carrying the patient mutation E97G (Senatore et al., 2021).

These data indicate an important role of the cAMP/UPS pathway in cilia dynamics and development, with potential pathogenic for ciliopathy disorders. Recently, heterozygous variants of catalytic subunits of PKA have been identified in individuals affected by a multiple congenital malformation syndrome characterized by cardiac defects, postaxial polydactyly and alterations of Shh pathway, suggesting a fundamental role of cAMP-PKA pathway in the pathogenesis of ciliopathies (Palencia-Campos et al., 2020).

Further studies are needed to better elucidate how UPS and autophagy are involved in the several signaling pathways that occurs at the primary cilia, mostly because understanding these molecular mechanisms could be useful for the study and the treatment of both ciliopathies and proliferative disorders that are often accompanied by alterations of ciliary dynamics (Eguether and Hahne, 2018).

## Concluding Remarks

Primary cilium is a sensory non-motile organelle that receives, transmits and integrates signaling inputs from extracellular microenvironment to intracellular compartment, playing a major role in key cellular activities. Derangement of ciliary activities contributes to the pathogenesis of human genetic and proliferative disorders. Cilium biogenesis, dynamics and functions are finely regulated by UPS and autophagy, and components of the proteolytic machineries are closely associated to ciliary compartment. By controlling localization and levels of ciliary proteins, UPS and autophagy regulate key aspects of cilium biology. These degradative systems are timely and spatially regulated by signaling pathways generated at the ciliary compartment or by distantly located receptors at non-ciliary membranes. Integration of signaling inputs and degradative pathways is expected to finely modulate ciliary activities in response to changes of extracellular microenvironment. Further studies are needed to identify additional, relevant ciliary substrates of UPS/autophagy pathways and to elucidate the molecular mechanisms by which autophagy and UPS differentially regulates ciliogenesis and dynamics in different cell types or under changed metabolic needs. Similarly, the differential regulation of cilium dynamics by signaling pathways activated at different intracellular sites, such as cAMP cascade, needs further investigation.

Understanding the complex regulation of primary cilium by UPS and autophagy machineries, and dissecting the signaling events controlling cilium biogenesis and dynamics will pave the way for the development of novel therapeutic approaches based on selective targeting of these degradative systems in genetic and proliferative disorders.

## Glossary

MIB1: mindbomb E3 ubiquitin ligase protein 1
PCM1: pericentriolar matrix protein 1
UBR5: ubiquitin protein ligase E3 component N-recognin 5
DAVs: distal appendages vesicles
BBS4: Bardet-Biedl syndrome 4
OFD1: oral-facial-digital syndrome 1
MYH9: myosin heavy chain 9
IFT20: intraflagellar transport protein 20
NR1H4: nuclear receptor subfamily 1 group H member 4
CSPP1: centrosome and spindle pole-associated protein 1
NEK10: never in mitosis A related kinase 10
SCF: Skip1-Cullin-F-box
KIF2c: kinesin family member 2c
Dvl2: dishvelled 2
HEF1: human enhancer of filamentation 1 protein
CUL3: cullin 3
PPARα: peroxisome proliferator-activated receptor
Shh: Sonic Hedgehog; Smo, smoothened
IFT27: intraflagellar transport protein 27
HERC4: HECT and RLD domain containing E3 ligase 4
SUFU: suppressor of fused protein
PTCH1: patched 1
GliA: activated Gli
NPHP5: nephrocystin 5
BBS11: Bardet-Biedl syndrome 11
MARCH7: membrane associated ring-CH-type finger 7
AurA: aurora A kinase
USP8: ubiquitin carboxyl-terminal hydrolase 8
EGFR: EGF receptor
AC: adenylate cyclase
PKA: protein kinase A
CHIP: C-terminus of Hsc70-interacting protein
Ub: ubiquitin
K48: lysine 48
K63: lysine 63
K11: lysine 11
LC3: microtubule-associated protein 1A/1B-light chain
RBR: RING-between-RING
